# Development and testing of the Geriatric Care Assessment Practices (G-CAP) survey

**DOI:** 10.1186/s12877-021-02073-5

**Published:** 2021-04-01

**Authors:** Justine L. Giosa, Paul Stolee, Paul Holyoke

**Affiliations:** 1 School of Public Health and Health Systems, University of Waterloo, 200 University Avenue West, Waterloo, ON N2L 3G1 Canada; 2 SE Research Centre, SE Health, 90 Allstate Parkway, Suite 300, Markham, ON L3R 6H3 Canada

**Keywords:** Assessment, Home care, Older adult, Survey, Interdisciplinary, interRAI, RAI-HC, Nurse, Occupational therapist, Physiotherapist

## Abstract

**Background:**

While the Resident Assessment Instrument-Home Care (RAI-HC) tool was designed to support comprehensive geriatric assessment in home care, it is more often used for service allocation and little is known about how point-of-care providers collect the information they need to plan and provide care. The purpose of this pilot study was to develop and test a survey to explore the geriatric care assessment practices of nurses, occupational therapists (OTs) and physiotherapists (PTs) in home care.

**Methods:**

Literature review and expert consultation informed the development of the Geriatric Care Assessment Practices (G-CAP) survey—a 33 question, online, self-report tool exploring assessment and information-sharing methods, attitudes, knowledge, experience and demographic information. The survey was pilot tested at a single home care agency in Ontario, Canada (*N* = 27). Test-retest reliability (*N* = 20) and construct validity were explored.

**Results:**

The subscales of the G-CAP survey showed fair to good test-retest reliability within a population of interdisciplinary home care providers [ICC2 (A,1) (*M ICC* = 0.58) for continuous items; weighted kappa (*M kappa* = 0.63) for categorical items]. Statistically significant differences between OT, PT and nurse responses [*M* t = 3.0; *M p* = 0.01] and moderate correlations between predicted related items [*M r* = |0.39|] provide preliminary support for our hypotheses around survey construct validity in this population. Pilot participants indicated that they use their clinical judgment far more often than standardized assessment tools. Client input was indicated to be the most important source of information for goal-setting. Most pilot participants had heard of the RAI-HC; however, few used it. Pilot participants agreed they could use assessment information from others but also said they must conduct their own assessments and only sometimes share and rarely receive information from other providers.

**Conclusions:**

The G-CAP survey shows promise as a measure of the geriatric care assessment practices of interdisciplinary home care providers. Findings from the survey have the potential to inform improvements to integrated care planning. Next steps include making adaptations to the G-CAP survey to further improve the reliability and validity of the tool and a broad administration of the survey in Ontario home care.

**Supplementary Information:**

The online version contains supplementary material available at 10.1186/s12877-021-02073-5.

## Background

Older adults want to remain in their own homes as long as possible, and meeting their often compounding physical, functional, cognitive, and psychosocial needs with home care services is a key priority for Canadian health care and health care systems internationally [1–4]. With the complexity of geriatric home care client needs and the number of different care providers potentially involved, a variety of information and data are required to plan and deliver effective home care services. How, when and who collects this information is very important to the experience of integrated care [5]. To prevent duplication, repetition and frustration, a common assessment approach is preferred over each care provider completing their own assessment. This allows for the development of a comprehensive picture of health care needs, while effectively reducing the demand on older adult home care clients and their family/friend caregivers to repeat their story and health history multiple times to different people [5–7].

A well-documented model for health care planning and delivery to older adults with complex health issues is the Comprehensive Geriatric Assessment (CGA). Often thought to be synonymous with specialized geriatric medicine, CGA emphasizes an interdisciplinary and multidimensional approach to assessment that requires all involved health care providers to input information on the functional, social and environmental factors related to an older individual’s health, in conjunction with their diagnoses [8, 9]. International evidence on CGA indicates it has been used in a variety of geriatric care settings across the continuum of care. It has been most well established for use in hospital settings, with studies reporting its ability to predict adverse events [10], lead to improved functional outcomes [11, 12] and decrease morbidity, mortality and hospital admissions [13–15]. The use of CGA in primary and community care has also been documented [8, 9]. Trials of CGA combined with multidimensional interventions for community-dwelling older adults have shown improvement in clients’ self-reported ability to complete activities of daily living [16, 17]. CGA has also been used by Mobile Geriatric Assessment Teams to coordinate the provision of targeted multidisciplinary primary care to rural-dwelling, and frail, older adults and has been applied in a preventive context for at-risk community-dwelling older people [18–20].

A key element of CGA is that comprehensive assessment and delivery of care are intended to be both integrated and carried out by point-of-care providers, yet there is little evidence on how this is practically achieved in the home care setting. interRAI is a collaboration of international researchers and practitioners in over 35 countries who have developed a suite of comprehensive assessment tools designed to support evidence-informed decision making across the continuum of care [21]. The Resident Assessment Instrument-Home Care (RAI-HC) is a standardized patient assessment tool designed to collect comprehensive patient information for care planning and collaborative decision-making by multiple providers in home care and is used in many countries around the world [22–27]. Since 2002, the RAI-HC has been mandated for use in Ontario, Canada to guide service allocation of government-funded home care services [28]. However, care coordinators have 14 days following patient admission to complete RAI-HC assessments and the data are not routinely shared in a useable format or applied by direct-service home care agencies in their delivery of services [28, 29]. Multiple providers from different health care disciplines are often involved in the direct care of older adults, but they work in isolation of each other in individual client homes and therefore individually collect the information they need to provide care [30–32].

The way the RAI-HC effectively combines cross-disciplinary information in a standard format makes it ideal to guide CGA practice in Ontario home care, yet the structure and organization of care in this sector may be impeding the opportunity for this tool to be used to its full capacity. Numerous layers of service provision and a lack of role clarity between assessment for service allocation and point-of-care planning in Ontario home care often result in multiple assessments for each client [33].

Nurses, occupational therapists (OTs) and physiotherapists (PTs) are the most common providers conducting patient assessments at the point-of-care in home care [34]. However, to date, their specific assessment and information sharing practices are largely unknown and undocumented. An understanding of the geriatric care assessment practices of individual providers is required to determine how to optimize individual provider contributions to CGA and care planning in this sector. More integrated care planning at the point-of-care has the potential to enhance both the quality and the experience of geriatric home care [35]. Consultation research to address this knowledge gap in home care is challenging as the geographic dispersion of providers and variable care schedules of clients make it difficult to coordinate and conduct face-to-face interviews and focus groups [36]. As an alternate approach, online surveys are an effective method to reach a broader group of people, and allow providers to participate at their convenience [37].

The purpose of this study was to develop and pilot test an online self-report survey tool to explore the geriatric care assessment practices of nurses, OTs and PTs in home care.

## Methods

### Survey development

The Geriatric Care Assessment Practices (G-CAP) survey was developed using multiple sources of information and guided by a multi-step approach recommended by Streiner et al. [38]:

1. Confirm there is no pre-existing survey tool

A scan of published and grey literature was completed to confirm there were no pre-existing tools for collecting data on the geriatric care assessment practices of point-of-care providers in home care.

2. Determine specificity of the tool

Informed by the background and scope of the project, the researchers determined that the G-CAP survey would focus on the geriatric assessment practices of nurses, OTs and PTs in home care. In accordance with Ontario’s Action Plan for Seniors the geriatric population was defined as any individual aged 65 years and older who was currently receiving home care for any health issue [39].

3. Consider homogeneity of the tool

Researchers hypothesized that the G-CAP survey items would be meaningful at the individual level and therefore would not be added together to generate a single composite score. However, the researchers planned to explore internal consistency (α) between subsets of seemingly related items to determine whether sub-scales existed within the tool. If present, this would indicate groups of effect indicators of sub-constructs related to the overall construct of geriatric assessment [38].

4. Determine the range of items to be included in the scale

As it is preferable in scale development to derive items from multiple sources, previous literature and expert opinion were used to create the item pool [38]. A scan of published and grey literature and current practices in CGA was completed to determine relevant geriatric care domains, standardized assessment tools and other items that should be explored in this type of survey. A group of clinical leaders from various disciplines involved in geriatric home care at a Canadian home care agency were also consulted to help formulate additional items for inclusion in the G-CAP survey.

A first draft of the survey was developed based on the candidate domains and items from the literature and clinical leadership group (see Additional file 1). To further refine the survey tool, a convenience sample of management, education and clinical experts in nursing, occupational therapy and physiotherapy (*N* = 7) were recruited to participate in key informant interviews where they were asked to review and confirm the candidate list of domains and items to be included in the survey and comment on face validity and content validity (relevance, representativeness and coverage of items). The key informants were also asked to review survey items for any ambiguous wording and comment on the overall length of the tool from a feasibility perspective [38]. All key informants provided written consent to participate in the interviews, which were audio-recorded and transcribed verbatim. Interview transcripts were thematically analyzed by two independent researchers using an inductive coding method and NVivo 10 software [40–42]. Each researcher completed a line-by-line analysis of the transcripts to code meaningful units of data, which were then brought together into categories that were labeled according to similarities in meaning. Categories were then compared and organized into themes related to survey tool validity and the adoption of a common assessment approach in home care [41, 42]. After completing their individual analyses, the two researchers came together to compare, contrast and finalize the themes.

5. Scaling the responses

Researchers determined that three different types of response options were needed to match the question types in the refined pool of survey items: 1) perceived frequency; 2) level of agreement; and 3) perceived importance. As these response options are bipolar in nature, they were scaled on a seven-point Likert type scale [38].

### Pilot testing

#### Reliability and validity

Test-retest reliability of the G-CAP survey for use with nurses, OTs and PTs in home care was explored to determine the stability of provider responses about their geriatric care assessment practices over time [38]. Point-of-care providers were asked to participate in the survey on two separate occasions, time one (T1) and time two (T2), which were separated by a period of 2 weeks. To determine if the G-CAP survey measures the intended geriatric care assessment constructs with nurses, OTs and PTs in home care, construct validity was explored. Hypotheses were generated and tested to explore expected differences (discriminative validity) and relationships (divergent and convergent validity) between various attributes of the survey and behaviours of respondents based on discussions with the clinical leadership group [38]. Discriminative construct validity was explored by testing the following hypotheses about differences between nurse, OT and PT responses:

- Rehabilitation therapists (OTs and PTs combined) will use measures of functional status/ activity and rest more often than nurses;
- Nurses will use measures of skin integrity more often than rehabilitation therapists;
- Rehabilitation therapists will assess mobility more often than nurses;
- Rehabilitation therapists will use measures of mobility more often than nurses; and
- OTs will use measures of the patient environment more often than PTs.

Convergent and divergent construct validity was explored by testing the following hypotheses about correlations between survey items:

- Years of experience will be positively correlated with having heard about the RAI-HC;
- Opinions that client assessment requires observation of a client in their home will be positively correlated with the use of observation and interview skills;
- Believing assessment involves conversations with health care providers will be positively correlated with sharing information; and
- Believing that standardized assessment tools are part of geriatric assessment will be negatively correlated with years of experience.

### Sample size

To make sure the analysis of test-retest reliability was appropriately powered, the hypothesis testing approach of Kraemer and Thiemann [43] was used to determine an appropriate sample size for G-CAP survey participants. To determine whether an “excellent” reliability of > 0.75 was significantly different from a “poor” reliability of 0.40, a target sample size of 21 participants at T1 and T2 was determined to be appropriate [43–45]. This sample size is also sufficient for detecting large correlations (> 0.5) [43, 46].

### Recruitment

Point-of-care nurses, OTs and PTs in four geographic areas within a single home care provider agency in Ontario made up the participant pool for this study. Inclusion criteria to participate in the research study included being actively registered with a professional college for one of the three disciplines of interest (nursing, occupational therapy or physiotherapy) in Canada, currently working as a point-of-care care provider in home care in Ontario, Canada and being able to read and write English. A convenience sampling strategy was employed until the target sample size was reached. T1 recruitment began with telephone information sessions between a researcher (JG) and clinical leaders within each of the four geographic areas. Following these information sessions, blast email messages were sent out by clinical leaders to approximately 290 frontline staff requesting their voluntary participation in the survey, providing a link to the online survey in SurveyMonkey and outlining a one-week deadline for participation. All survey participants were provided with necessary study information at the beginning of the survey and consent was implied from their voluntary submission of the survey.

Point-of-care providers who decided to participate in the survey were asked to provide their email addresses at the end of T1 survey completion. Within 1 week, a researcher (JG) emailed each T1 survey participant directly, inviting them to participate in the survey at T2, and providing them with a one-week deadline to do so. This deadline was to ensure that both T1 and T2 survey completion took place within a 14-day period; an optimal time frame for test-retest reliability [38]. Up to two reminder emails were sent to each participant to complete the survey, after which point if they had not participated, it was assumed that they had decided to withdraw from the study. As participants completed the survey electronically, they did not have access to their T1 responses when completing the survey at T2. Participant responses were de-identified after T2 survey completion and each participant was assigned a unique study identification number for the purposes of linking T1 and T2 responses together.

Participants were not paid for their time to complete the survey at T1 or T2, but in recognition of their efforts, they were given the option to enter their name into a draw for one of four gift cards ($50 CAD each) if they completed the survey at both T1 and T2.

### Data analysis

Participant survey responses at T1 were used to provide demographic information and to complete construct validity analyses; data from T1 and T2 were used to analyze test-retest reliability. All skipped frequency questions were coded as “never”, and all skipped agreement or importance questions were coded as “neutral”.

Statistical analyses were completed using IBM SPSS 20.0 software, beginning with descriptive statistics [47]. First, internal consistency (*α*) was explored for groups of related categorical items. Cronbach’s alpha values less than 0.5 were considered unacceptable, between 0.51 and 0.60 were considered poor, between 0.61 and 7.0 were considered acceptable, between 0.71 and 0.90 were considered good and greater than 0.90 were considered excellent [48]. For groups of items with α > 0.61, a single Intra-Class Correlation Coefficient (ICC2, A1) was calculated to determine test-retest reliability for these potential sub-scales of related items [38]. The test-retest reliability of individual categorical items of the G-CAP survey was evaluated using weighted kappa coefficients with quadratic weights. Following the guidelines suggested by Fleiss [44], reliability values below 0.40 were considered poor, between 0.41 and 0.75 were considered fair to good and > 0.75 were considered excellent. Discriminative construct validity was evaluated by comparing mean results using a two-tailed independent samples t-test statistic with a 5% level of significance (*α* = 0.05) for various hypotheses about differences between disciplines. Convergent and divergent construct validity was tested by calculating Pearson product moment correlations to test various theories about relationships between items in the G-CAP survey. Following the guidelines suggested by Cohen [46], correlations of 0.1 were considered small, of 0.3 were considered moderate, and of 0.5 were considered large.

## Results

### The G-CAP survey

An initial scan of published and grey literature identified various classifications of care domains relevant to CGA. Table 1 illustrates some examples of these different classifications.

**Table 1:** Examples of CGA assessment domain classifications reported in the literature

Guthrie et al., 2014 [28]	Elsawy & Higgins, 2011 [49]	Cobb, Duthie & Murphy, 2002 [50]	Stauder et al., 2010 [51]	Fleming & Scamehorn, 2006 [52]	Gallo et al., [53]	Stolee, 2010 [27]
•Functional ability •Communication •Pain •Cognition •Mood •Service use	•Functional ability (Activities of Daily Living (ADLs); Independent Activities of Daily Living (IADLs)) •Physical health (disease screening, nutrition, vision, hearing, continence, balance and fall prevention, osteoporosis, polypharmacy) •Cognition and mental health (depression, dementia) •Socio-environmental circumstances	•Physical (functional status, nutrition, vision, hearing) •Cognitive (dementia) •Psychological (depression, anxiety) •Social (personal support, caregiver burden, advance directives, abuse) •Driving (assess risks)	•General functioning in everyday life •Comorbidities •Nutritional status •Cognition •Health-related quality of life •Social support	•FEEBLE Forgetful Eyes Ears Brown bag of medications Leaking (continence) Eat •FALLERS Fall ADLs Lonely Living Expectations Rest Specialists •ARE Advanced directives Ride (driving) ED visits •FRAIlL Family Religion Access Income Lifestyle	•Functional ADLs/ IADLs •Cognition •Depression •Social and economic issues •Substance use •Driving	•Physical function •Cognitive function •Self-rated health •Psychosocial function •Healthcare use •Other

Consideration of these various conceptualizations of CGA domains in terms of their frequency of inclusion in the literature, relevance to home care, research and interdisciplinary practice led to defining a list of initial domains and items to consider for inclusion in the G-CAP survey (see Table 2). Additional academic and grey literature searching and consultation with the clinical leadership group led to refinement of the domains and item pool for inclusion in the survey, including the addition of eight standardized assessment tools, items related to opinions, use and knowledge of the RAI-HC and clinician observation and interview skills (see Table 2).

**Table 2:** Development of domains and items to be included in the survey

Source of input	Domains	Items
Literature (academic & grey)	•Cognition and mood •Pain •Wounds (skin) •Function •Mobility •Environment •Quality of life •Social support •Financial situation •Demographics	•50 standardized assessment tools
Clinical Leadership Group	•RAI-HC	•8 additional standardized assessment tools •Observation and interview skills •Opinions •Use •Knowledge/ awareness
Clinical Expert Key Informants	•Cognition and mood •Pain •Skin integrity •Functional status/ activity and rest •Mobility/ balance/ ambulation •Safety (environment, abuse risk and fall risk) •Medication management •Quality of life •Resources (social and financial) •Interdisciplinary collaboration	•9 additional standardized assessment tools •Attitudes •Experience

Key informant interviews indicated good face validity for the proposed survey domains and items. All key informants indicated that they believed the survey domains and items appeared to be assessing the geriatric care assessment practices of point-of-care home care providers and felt that the data provided would be valuable. For example, one expert indicated: “This is nice…it is nice. I think it is nice. It will be interesting to see what you are going to get…I think it will be really interesting to see what comes out of it”.

In terms of content validity, key informants were generally supportive of the items included in the survey; however, they suggested a reclassification of some of the survey domains using language they felt would be better understood by home health care providers. Key informants suggested nine additional standardized assessment tools that should be included in the survey (see Table 2).

Clinical expert key informants also discussed various barriers and facilitators to adopting an interdisciplinary common assessment approach in home care (see Table 3). These perceptions of barriers and facilitators informed the inclusion of additional survey items related to attitudes towards assessment, and experiences with interdisciplinary collaboration.

**Table 3:** Expert opinions regarding barriers and facilitators for moving to common assessment approaches in home care

Barriers	Facilitators
Competing care priorities across disciplines	Identification and prioritization of client goals
Too many standardized assessment tools available	Knowing what data are needed by all health care providers
Health care providers working in isolation of each other	Interdisciplinary collaboration
No access to data collected by other health care providers	Leveraging technology for information-sharing

Experts indicated that the survey was quite long, although they also agreed that all the items were necessary for a thorough exploration of geriatric assessment practices. This prompted the decision to include automatic skip patterns in the online survey so that participants would not spend time responding to questions in an area that was not applicable to their individual geriatric assessment practices.

The final version of the G-CAP survey included 33 questions related to the following five areas: 1) Assessment methods; 2) Attitudes toward assessment; 3) Perceptions of the RAI-HC; 4) Interdisciplinary collaboration; and 5) Demographic information (see Additional file 2).

### Demographics

A total of 27 out of ~ 290 health care providers (9.3%) who were emailed the survey, participated at T1. Of these 27 participants, 20 (74.1%) subsequently participated in the survey at T2. Participation took place between September 1, 2014 and November 30, 2014. Participants were mostly female (96.3%) and ranged in age from 23 to 75 years (*M* = 42.6, *SD* = 13.8), with an average of 15.6 years of experience in their respective disciplines (*SD* = 12.7, Range: 1–53). More than half of the participants (55.6%) had been working in home care for at least five years, with one-third (33.3%) having worked in the sector longer than 10 years. Most participants had experience working in other health care sectors, with 70.4% having previously worked in a hospital and 51.9% in long-term care. Most participants (88.9%) indicated that more than half of their home care clients are over the age of 65 years. The characteristics of participants are displayed in Table 4.

**Table 4:** Characteristics of survey participants

Characteristic		All providers (***N*** = 27)	Nurses (***n*** = 12)	OTs (***n*** = 8)	PTs (***n*** = 7)
Age^a^ Mean (SD) (Range)		42.6 (13.8) (23–75)	41.1 (14.9) (23–67)	46.4 (15.2) (30–75)	41.0 (11.6) (29–60)
Gender *n*		Female *26*, Male *1*	Female *12*	Female *8*	Female *6* Male *1*
Years in practice Mean (SD) (Range)		15.6 (12.7) (1–53)	10.2 (9.3) (1–28)	22.6 (15.4) (6–53)	16.9 (11.6) (7–37)
Working in home care *n* (%)	< 1 year:	*5* (18.5)	*4* (33.3)	*0* (0)	*1* (14.3)
	1–5 years:	*7* (25.9)	*2* (16.7)	*1* (12.5)	*4* (57.1)
	6–10 years:	*6* (22.2)	*2* (16.7)	*3* (37.5)	*1* (14.3)
	> 10 years:	*9* (33.3)	*4* (33.3)	*4* (50.0)	*1* (14.3)
Working in other sectors *n* (%)	Hospital	*19* (70.4)	*9* (75.0)	*6* (75.0)	*4* (57.1)
	LTC^b^	*14* (51.9)	*9* (75.0)	*1* (12.5)	*4* (57.1)
	Rehab^c^	*6* (22.2)	*1* (8.3)	*3* (37.5)	*2* (28.6)
	Palliative	*5* (18.5)	*2* (16.7)	*2* (25.)	*1* (14.3)
	Private	*11* (40.7)	*2* (16.7)	*3* (37.5)	*6* (85.7)
Clients over 65 years *n* (%)	< 25%	*0* (0)	*0* (0)	*0* (0)	*0* (0)
	26–50%	*3* (11.1)	*2* (16.7)	*1* (12.5)	*0* (0)
	51–75%	*12* (44.4)	*7* (58.3)	*3* (37.5)	*2* (28.6)
	76–100%	*12* (44.4)	*3* (25.0)	*4* (50.0)	*5* (71.4)

^a^Two participants did not indicate their age

^b^*LTC* Long-Term Care

^c^ *Rehab* Inpatient Rehabilitation

### Reliability

ICC2 (*A,1*) coefficients indicate fair to good test-retest reliability, for most groups of related categorical items and excellent test-retest reliability for one group of related categorical items comprising potential sub-scales of the G-CAP survey within a population of interdisciplinary home care providers (*M* = 0.58) (see Table 5).

**Table 5:** Test-retest reliability for groups of related categorical items (potential-subscales)

G-CAP Survey Section	Potential Sub-Scale Name	Questions (items)	Internal Consistency ***(****α****)***	***ICC 2 (A,1)*** (95% Confidence Interval)
Methods of Assessment	Assessment of Geriatric Care Domains	1, 3, 5, 7, 9, 11, 13, 15, 17	*0.91*	*0.57* (0.46–0.66)
	Use of Clinical Observation and Interview Skills	2i, 4i, 6d, 8 l, 10 l, 12f, 14 h, 16c, 18n	*0.89*	*0.41* (0.0–1.0)
Attitudes Toward Client Assessment in Home Care	Holistic Assessment Practices	19 a-l	*0.72*	*0.62* (0.53–0.69)
Perceptions of the RAI-HC Assessment Tool	Use of RAI-HC	21 a-c	*0.74*	*0.78* (0.66–0.86)
Interdisciplinary Collaboration	Collaborative Goal-Setting	23 a-g	*0.82*	*0.52* (0.39–0.62)
	Interdisciplinary Information-sharing	25 a-e	*0.76*	*0.53* (0.37–0.66)

Mean weighted kappa coefficients indicate fair to good test-retest reliability, on average, for individual categorical items of the G-CAP survey within a population of interdisciplinary home care providers (*M kappa* = 0.63) (see Table 6).

**Table 6:** Test-retest reliability for individual categorical items

G-CAP Survey Section	Questions (items)	***Mean Weighted Kappa Coefficient*** (Range)
Methods of Assessment	2a, 2b, 4a, 4b, 4c, 4e, 4f, 6a, 8a, 10e, 12b^a^	*0.64* (0.30–0.98)
Perceptions of the RAI-HC Assessment Tool	20	*0.66* (0.37–0.95)
	22 a-f	*0.56* (0.08–0.97)
Interdisciplinary Collaboration	24 a-d	*0.65* (0.46–0.75)
Demographic Information	28, 30, 31, 33^b^	*0.62* (0.43–0.90)

^a^Only questions about tools that were rated to be used more than almost never (> 2 on a 7 point scale) were included in the analysis

^b^Question 32 was excluded due to a glitch in the online question format and given that all providers were from the same home care agency

### Validity

Significant two sample t-test statistics (*p* < 0.05, two-tailed) confirmed the hypothesized differences among nurse, OT and PT responses. Table 7 depicts the t-test scores that support each hypothesis about differences between these groups (*M* t = 3.0; *M p* = 0.01), which demonstrates preliminary discriminative construct validity for use of the G-CAP survey with interdisciplinary home health care providers.

**Table 7:** Discriminative construct validity for use of the G-CAP survey with interdisciplinary home health care providers

Hypotheses about differences between groups	G-CAP item	Mean	t test value (***p*** value)
Rehabilitation therapists (OTs and PTs combined) will use measures of functional status/ activity and rest more often than nurses	Functional Independence Measure (FIM)	Therapist *M = 3.4*	4.0 (0.001)
		Nurse *M = 1.0*	
	Functional Reach Test	Therapist *M = 1.6*	2.9 (0.012)
		Nurse *M = 1.0*	
Nurses will use measures of skin integrity more often than rehabilitation therapists	Braden Scale for Pressure Sore Risk	Nurse *M = 5.0*	3.6 (0.002)
		Therapist *M = 2.4*	
Rehabilitation therapists will assess mobility more often than nurses	Assessment of mobility/ balance/ ambulation	Therapist *M = 6.5*	2.3 (0.037)
		Nurse *M = 5.1*	
Rehabilitation therapists will use measures of mobility more often than nurses	Berg Balance Scale	Therapist *M = 2.5*	3.5 (0.003)
		Nurse *M = 1.1*	
	Timed Up and Go Test (TUG)	Therapist *M = 2.7*	3.2 (0.004)
		Nurse *M = 1.0*	
OTs will use measures of the patient environment more often than PTs	SAFER-HOME	OTs *M = 2.8*	1.8 (0.013)
		PTs *M = 1.0*	

Pearson’s product moment correlation coefficients *(r)* confirmed expected convergent and divergent relationships between survey items and demographic information. Table 8 details the correlation coefficients for each hypothesis tested, with moderate correlation values, on average (*M* r = |0.39|), which demonstrates preliminary convergent and divergent construct validity for use of the G-CAP survey with interdisciplinary home health care providers.

**Table 8:** Convergent and divergent construct validity for use of G-CAP survey with home health care providers

Hypotheses	G-CAP Questions (items)	Pearson’s correlation *r*
Years of experience in general and in home care will be positively correlated with having heard about the RAI-HC	29 and 20	0.27
	30 and 20	0.25
Opinions that client assessment requires observation of a client in their home will be positively correlated with the use of individual observation and interview skills in each domain	19f and 2i	0.33
	19f and 4i	0.33
	19f and 6d	0.36
	19f and 8 l	0.73*
	19f and 10 l	0.63*
	19f and 12f	0.60*
	19f and 14 h	0.39**
	19f and 16b	0.44**
	19f and 18n	0.44*
Believing assessment involves conversations with providers within and across disciplines will be positively correlated with sharing and receiving information within and across disciplines	19d and 25a	0.24
	19d and 25d	0.34
	19e and 25b	0.27
	19e and 25e	0.27
Believing that standardized assessment tools are part of geriatric assessment will be negatively correlated with the number of years in practice and in home care	19a and 29	−0.43*
	19a and 30	−0.36

*p < 0.01 (two-tailed)

**p < 0.05 (two-tailed)

### Preliminary survey findings

Pilot survey data point to five notable findings regarding the geriatric care assessment practices of nurses, OTs and PTs in home care.

#### Survey participants use their own clinical observation and interview skills far more often than any standardized tools for geriatric assessment

Participants indicated that they use their own observation and interview skills to assess each of the nine geriatric care domains included in the G-CAP survey (*M* = 5.6/7, *SD* = 2.1, Range: 1–7) significantly more often than any standardized assessment tools (*M* = 1.7/7, *SD* = 1.6, Range: 1–7). The only standardized assessment tools that participants indicated they used more than “almost never” (> 2 on a 7 point scale), on average, were the Numeric Pain Rating Scale (NPRS), which is used often (*M* = 5.0/7, *SD* = 2.4, Range: 1–7), the Verbal Rating Scale for pain, which is used often (*M* = 5.0/7, *SD* = 2.4, Range: 1–7) and the Braden Scale for Predicting Pressure Score Risk, which is rarely used (*M* = 3.4/7, *SD* = 2.5, Range: 1–7).

#### The majority of survey participants had heard of the RAI-HC, but do not actually use it

59.3% of the survey participants had previously heard about the RAI-HC, yet, on average, never use it to conduct comprehensive assessments of older home care clients (*M* = 1.66/7, *SD* = 1.7, Range: 1–6).

#### Participants said that the client input is the most important source of information for goal-setting

On average, participants rated input from the client as the most important (*M* = 6.7/7, *SD* = 0.45, Range: 6–7) for setting individual client goals. Participants consistently rated the assessment data that others collect (*M* = 5.9/7, *SD* = 0.78, Range: 4–7) as well as the professional opinion of other health care providers as less important (*M* = 5.9/7, *SD* = 0.80, Range: 4–7) when establishing these goals.

#### Participants agreed that they could use client information collected by other health care professionals but also agreed that they need to conduct client assessments themselves in order to provide care

While participants strongly agreed that they could use patient information collected by other health care professionals (*M* = 6.0/7, *SD* = 0.83, Range: 4–7), they also somewhat agreed that they must conduct client assessments themselves in order to provide care to clients (*M* = 5.7/7, *SD* = 1.3, Range: 1–7 on a 7 point scale).

#### Participants only sometimes share, and rarely receive assessment information from other health care providers

Participants indicated they only sometimes share client information with other health care providers in their discipline (*M* = 4.2/7, *SD* = 1.6, Range: 2–7) or outside of their discipline (*M* = 4.3/7, *SD* = 1.4, Range: 1–4). While participants sometimes indicated they receive client information from other health care providers in their discipline (*M* = 4.0/7, *SD* = 1.4, Range: 1–7), they rarely receive client information from other health care providers outside of their discipline (*M* = 3.7/7, *SD* = 1.3, Range: 1–7).

## Discussion

### Reliability and validity of the geriatric care assessment practices (G-CAP) survey

The G-CAP survey showed fair to good test retest reliability according to the Fleiss criteria [44]. However, it is important to note that these criteria are not specific to ICC, kappa and correlation values and are routinely used to interpret many different types of reliability coefficients in the literature. Therefore, setting reliability cut-off values has been reported to be a fairly arbitrary, although common practice, in the development of novel measurement tools and scales [38, 54]. The author Nunnally [55], however, adds a critical distinction for interpreting psychometric data, based on the purpose of the tool that is being developed. If the tool is being used for research purposes, a reliability coefficient of at least 0.70 is suggested; whereas, tools being used for clinical decision-making should have reliability values of at least 0.90 [55].

As the G-CAP survey was developed specifically for research purposes, there is room for some improvement in test-retest reliability. Participant responses were almost exclusively at the high end of the scale (*M* = 5.6/7), for the frequency of assessment on each care domain, while their responses for the frequency of utilizing standardized assessment tools was substantially lower (*M* = 1.7/7). Based on these results, modification of the scales to better distinguish between respective ceiling and floor effects would enhance reliability and the ability to discriminate between more nuanced positive and negative responses [38]. Changing the 7-point Likert type scale to a 5-point Likert type scale is predicted to improve scale reliability and shifting the neutral point of the scale depending on the question is predicted to improve the ability of the scale to discriminate between positive and negative responses [38]. These changes will be made prior to the broad scale administration of the G-CAP survey. Further, as reliability is context-specific, Streiner et al. [38] suggest that it tends to increase when a tool is administered in a more heterogeneous population, which is planned for the next phase of this research when the G-CAP survey is administered to a wider group of home care nurses, OTs and PTs.

Statistically significant differences between OT, PT and nurse responses and moderate correlations between predicted related items of the G-CAP survey tool provide preliminary support for our hypotheses around survey construct validity in this population. Modifications to the tool as described above and broader administration of the G-CAP survey will provide additional opportunities to explore its validity for use with interdisciplinary home care providers.

### Exploring the geriatric care assessment practices of nurses, OTs and PTs in home care

Survey participants said they use their clinical observation and interview skills far more than any standardized assessment tools when conducting geriatric assessments at the point-of-care in home care. Previous literature supports the use of clinical judgment in geriatric care, especially in predicting falls risk [56, 57]. One study found that clinical judgment was more accurate than traditionally used falls-risk assessment tools, although less sensitive [58]. Clinical judgment has also been shown to be more effective than standardized assessment in predicting frailty in geriatric patients with cancer [59]. However, standardized assessment has been found to be superior to clinical judgment in other areas of geriatric care, including functional assessment of cognition and ADLs, particularly in predicting more moderate impairments in function that could be targeted with earlier intervention and identifying frailty [60–62]. Further exploration of the individual and combined use of standardized tools and clinical judgement is needed to support a CGA type of assessment approach in home care.

Only 59.3% of surveyed home health care providers had previously heard about the RAI-HC. Of these participants, most also indicated that they never use the RAI-HC themselves to collect data about geriatric clients to plan and provide care. These results further illuminate the previously cited disconnect between system level assessment for the purposes of service allocation and point-of-care assessment for the purposes of real-time care delivery in Ontario home care [28]. Further, participants indicated that they use very few other standardized assessment tools, which is potentially indicative that they do not believe there to be a more appropriate alternative to the RAI-HC as a comprehensive standardized assessment at the point-of-care in geriatric home care. This suggests that the perceived potential of the RAI-HC is under-realized and supports the need to further explore the applicability of the RAI-HC in point-of-care assessment to foster real-time care planning.

Survey participants’ opinions regarding the priority of information sources for individual goal-setting indicate input from the client as most important. While participants’ prioritization of client input in goal-setting is aligned to current best practices in shared-decision making and person- and family-centred care for individual interactions between clients and providers, their responses are also reflective of the need to improve interdisciplinary collaboration in geriatric home care [63–67]. Participants indicated they only sometimes share and rarely receive client assessment information from other health care providers and that professional opinion and assessment data from other health care providers are the least important sources of information for client goal-setting. Additionally, 96.2% of participants indicated that they can make use of client data collected by other health care providers, but 85.1% of participants also said that they must conduct the patient assessment themselves to be able to provide care. These findings are in contrast to defined optimal collaborative practice, which Curran [68] says:

…involves the continuous interaction of two or more professionals or disciplines organized into a common effort to solve or explore common issues, with the best possible participation of the patient. Collaborative practice is designed to promote the active participation of each discipline in patient care. It enhances patient- and family-centred goals and values, provides mechanisms for continuous communication among caregivers, optimizes staff participation in clinical decision-making within and across disciplines and fosters respect for disciplinary contributions of all professionals. (p.1)

Further exploration is required into mechanisms for consistent and efficient communication and information-sharing between providers at the point-of-care in home care [69, 70].

### Strengths and limitations

This study has several strengths. To our knowledge, this is the first study to systematically explore the geriatric care assessment practices of point-of-care home care providers using survey methods and the G-CAP survey is the first tool of its kind. Another study strength includes the psychometric testing of the G-CAP survey tool, as the results indicate preliminary support for use of the instrument to explore the geriatric assessment practices of interdisciplinary point-of-care providers in home care and therefore may be useful for exploring geriatric care assessment practices of interdisciplinary providers in other geographies and care settings.

This study also has several limitations. First, the data represent a pilot implementation of the G-CAP survey and are only reflective of health care provider views in three disciplines in a single direct-service home care agency. However, the representation of nurses (*n* = 12), OTs (*n* = 8) and PTs (*n* = 7) in the study sample is reflective of the representation of these disciplines within home care in Ontario at the time of data collection. In 2010, there were 125,844 nurses working in Ontario and the community care sector employed 18.4% of these nurses; in 2011, there were 4506 occupational therapists working in Ontario, with 31.1% working in the community sector; and in 2009, there were 6391 physiotherapists working in Ontario, with 14.8% working in the community sector [71–73]. Further, the study sample represents four different geographic locations across Ontario. Additional research is required to explore the geriatric care assessment/client observation and information-sharing practices of other relevant disciplines within home care, including social workers, speech-language pathologists and personal support workers.

Another potential limitation of this study is that the data were collected in 2014. However, as the overall structure of the Ontario home care system has remained largely unchanged since that time, the findings are believed to be relevant to current care practices. A recent study on the use of home care assessment data in the home care sector confirms this relevance, indicating that these data are both undervalued and underutilized for evidence-informed decision-making across the sector [74].

Another limitation in the study methods was the low response rate to the G-CAP survey (9.3%), which might be attributed to the busy schedules of point-of-care providers, the length of the survey, lack of personalization in email administration or lack of direct remuneration. Methods were chosen to test an efficient approach for reaching large numbers of health care providers across the province, which is required in the next phase of this work where broad administration of the G-CAP survey will occur. The positive response rate following the researcher’s in-person promotion of the survey within a single OT team indicates the need for additional personalization of the survey experience to boost response rates in future stages [75].

## Conclusions

The newly developed G-CAP survey tool shows promise as a measure of the geriatric care assessment practices of interdisciplinary home health care providers.

Preliminary data indicate that point-of-care geriatric assessment in home care by nurses, OTs and PTs is heavily focused on clinical observation and interview skills, with limited use of the RAI-HC or any standardized assessment tools to collect client information. Although there is good intention to set and work towards common person-and family-centred goals by individual providers, limited information-sharing occurs between providers, both within and across disciplines.

Pilot results point to the potential to integrate RAI-HC data collected for service allocation at the system level with clinical judgment and assessment data collected by point-of-care providers to reflect a more CGA-type approach. Next steps include making adaptations to the G-CAP survey to further improve the reliability and validity of the tool and a broad administration of the G-CAP survey across multiple home care service provider agencies in Ontario. Results will be used to inform improvements to integrated geriatric care planning through improved documentation and standardization of clinical assessment practices using validated tools and sharing and using this information across the care team. A more seamless geriatric care planning approach that is consistent with the principles of CGA has the potential to transcend discipline, agency and system boundaries to achieve more efficient and integrated delivery of geriatric home care.

## Supplementary Information

**Additional file 1.** Draft G-CAP Survey for Clinical Expert Key Informant Consultation.

**Additional file 2.** Pilot Version of the G-CAP Survey

## Data Availability

The datasets generated and/or analyzed during the study are not publicly available due to them containing information that could compromise research participant privacy/consent. Anonymized data are available from the corresponding author on reasonable request.

## Abbreviations

RAI-HC: Resident Assessment Instrument-Home Care
OT: Occupational Therapist
PT: Physiotherapist
ICC: Intraclass Correlation Coefficient
CGA: Comprehensive Geriatric Assessment
T1: Time One
T2: Time Two
ADLs: Activities of Daily Living
IADLs: Instrumental Activities of Daily Living
