# Supplementary material for: Development and testing of the Geriatric Care Assessment Practices (G-CAP) survey
Source: BMC Geriatr. 2021 Apr 1;21:220. doi: 10.1186/s12877-021-02073-5 (PMC8015173; doi:10.1186/s12877-021-02073-5)
Supplement: Supplementary file 1 — Additional file 1. Draft G-CAP Survey for Clinical Expert Key Informant Consultation. [file 12877_2021_2073_MOESM1_ESM.docx]

**Additional file 1- Draft G-CAP Survey for Clinical Expert Key Informant Consultation**

**Section 1: Background**

First we would like to get to know you a little bit better by asking you a few questions about your role as a health care provider.

1. What is your professional designation? (Please choose from the list below)

Registered Nurse

Registered Practical Nurse

Occupational Therapist

Physiotherapist

Speech Language Pathologist

Prefer not to say

2. In what year did you receive this professional designation? (Please type response in the space below)

__________________________________________

Prefer not to say

3. How long have you been working in the home care sector? (Please choose from the list below)

Less than one year

1-5 years

6-10 years

Greater than 10 years

Prefer not to say

4. What service provider agency do you currently work for? (Please type response in the space below)

__________________________________________

Prefer not to say

**Section 2: Information Needs**

Next, we would like to ask you a few questions about the information you need as a [nurse/PT/OT] to provide care to elderly home care clients. Please answer these questions according to your individual role as either a nurse, PT or OT.

5. Based on your experience as a [nurse/OT/PT] please describe your understanding of the purpose of patient assessment. (Please type response in the space below)

**__________________________________________________________________________________________________________________________________________________________________________________________________________________________________________**

Prefer not to say

6. Please list the three most important pieces of information you need to learn about an **elderly** client on your **first home care visit** with them. (Please type response in the space below)

1.____________________________________________________________________________

2.____________________________________________________________________________

3.____________________________________________________________________________

Prefer not to say

7. How do you usually collect these important pieces of information during or prior to your first home care visit with an elderly client? (Please choose all that apply from the list below)

Standardized assessment tool(s)

Non-standardized assessment tool(s) developed by you or your organization

Conversation with client

Conversation with client’s family member(s)

Review of client’s chart

Discussion with other health care providers in my discipline

Discussion with other health care providers outside my discipline

Other (please list) ___________________________________________________________

Prefer not to say

8. Is there any particular information you need about an elderly client in order to provide care to them has a [nurse/PT/OT] that you feel only a [nurse/PT/OT] can collect?

Yes

No

Prefer not to say

9. [if answer to question 8 is yes] Please indicate what information only your discipline can collect (Please type response in space below): ____________________________________________________________________________________________________________________________________________________________

**Section 3: Assessment Tools**

Next, we would like to ask you a couple of questions to learn more about your assessment practices.

10. Please indicate which assessment tools (standardized and non-standardized) that you routinely use to assess older home care clients and make decisions about their care needs. (Please check all that apply)

Cognition and Mood

Montreal Cognitive Assessment (MoCA)

Mini Mental State Examination (MMSE)

Geriatric Depression Scale (GDS)

Centre for Epidemiological Studies Depression Scale (CES-D)

Glasgow Coma Scale

The Delirium Index

The Confusion Assessment Method (CAM)

Other measure(s) related to cognition and mood (Please List Below) ____________________________________________________________________________________________________________________________________________________________________________________________________________________________________________________________________________________________________________________________________________________________________________________________________________________________________________________________________________________

Pain

Brief Pain Inventory

Numeric Rating Scale (Pain)

Verbal Rating Scale (Pain)

Visual Analogue Scale (Pain)

Facial Grimace and Behaviour Checklist Flowcharts (Pain)

McGill Pain Questionnaire

Other measure(s) related to pain (Please List Below) ____________________________________________________________________________________________________________________________________________________________________________________________________________________________________________________________________________________________________________________________________________________________________________________________________________________________________________________________________________________

Wounds

Braden Scale for Predicting Pressure Sore Risk

Bates-Jensen Wound Assessment Tool (BWAT)

Pressure Ulcer Scale for Healing (PUSH)

Other measure(s) related to wounds (Please List Below) ____________________________________________________________________________________________________________________________________________________________________________________________________________________________________________________________________________________________________________________________________________________________________________________________________________________________________________________________________________________

Function

Functional Independence Measure (FIM)

Katz Index of Independence in Activities of Daily Living

Barthel Index

Borg Rating Scale of Perceived Exertion

Functional Reach Test

Reintegration to Normal Living Index

Assessment of Motor Skills and Process

Other measure(s) related to function (Please List Below) ____________________________________________________________________________________________________________________________________________________________________________________________________________________________________________________________________________________________________________________________________________________________________________________________________________________________________________________________________________________

Mobility

Community Balance and Mobility Scale

Lower Extremity Functional Scale

Berg Balance Scale

Short Form Berg Balance Scale 3 Point

Timed Get Up and Go Test

Timed-Stands Test

Five Times Sit to Stand Test

Walk Test—2 minute, 6 minute, 12 minute, self-paced, shuttle

Gait speed

Activities-Specific Balance Confidence Scale

Other measure(s) related to mobility (Please List Below) ____________________________________________________________________________________________________________________________________________________________________________________________________________________________________________________________________________________________________________________________________________________________________________________________________________________________________________________________________________________

Environment

SAFER-HOME

Falls Risk Assessment Tool (FRAT)

Falls Risk for Older People in the Community (FROP-Com)

Indicators of Abuse (IOA)

Caregiver Abuse Screen (CASE)

Other measure(s) related to environment (Please List Below) ____________________________________________________________________________________________________________________________________________________________________________________________________________________________________________________________________________________________________________________________________________________________________________________________________________________________________________________________________________________

Quality of Life

Community Integration Questionnaire II

Life Satisfaction Questionnaire 9

(EuroQoL-5D) European Quality of Life Scale

Health Utilities Index (HUI Mark 2/3)

Nottingham Health Profile

SF-12 (12-item short-form health survey)

SF-36 (Medical Outcomes Study 36-item short-form health survey)

Other measure(s) related to quality of life (Please List Below) ____________________________________________________________________________________________________________________________________________________________________________________________________________________________________________________________________________________________________________________________________________________________________________________________________________________________________________________________________________________

Social Support

Multidimensional Scale of Perceived Social Support

Social Support Inventory (SSI)

General Social Survey (GSS)

Index of Social Support

Practitioner Assessment of Network Type (PANT)

Personal Resource Questionnaire (PRQ85)

The MOS Social Support Survey

The RAND Social Health Battery

Assessment of Perceived Loneliness

Assessment of Social Isolation

Social Support Questionnaire

Interpersonal Support Evaluation List (ISEL)

Other measure(s) related to social support (Please List Below) ____________________________________________________________________________________________________________________________________________________________________________________________________________________________________________________________________________________________________________________________________________________________________________________________________________________________________________________________________________________

Financial Situation

Semi-Structured Clinical Interview for Financial Capacity; SCIFC

Other measure(s) related to financial situation (Please List Below) ____________________________________________________________________________________________________________________________________________________________________________________________________________________________________________________________________________________________________________________________________________________________________________________________________________________________________________________________________________________

Other measures that do not fit in above categories (Please List Below)

____________________________________________________________________________________________________________________________________________________________________________________________________________________________________________________________________________________________________________________________________________________________________________________________________________________________________________________________________________________

11. Do you have experience using the Inter-RAI Home Care Assessment Tool (RAI-HC)?

Yes

No

Prefer not to say

12. [If answer to question 11 is yes] Please indicate in what capacity you have used the RAI-HC tool or (Please choose all that apply from the list below)

To conduct a client assessment

As part of a research study

Other: (please list) ________________________________________________________________________________________________________________________________________________________________________________________________________________________

Prefer not to say

13. Do you have experience using RAI-HC data or associated Clinical Assessment Protocols (CAPs)?

Yes

No

Prefer not to say

14. [If answer to question 13 is yes] Please indicate in what capacity you have used RAI-HC data or associated Clinical Assessment Protocols (CAPs)?

To plan client care

As part of a research study

Other: (please list)

________________________________________________________________________________________________________________________________________________________________________________________________________________________

Prefer not to say

**Section 4: Goal Setting**

Next, we would like to ask you a few questions around client goal-setting.

15. Are you involved in setting individualized goals with elderly home care clients related to the [nursing/OT/PT] care you provide?

Yes

No

Prefer not to say

16. [If answer to question 15 is yes] Please rank from 1-5 (1 being most important and 5 being least important) the importance of the following information sources when setting individual client goals related to the [nursing/OT/PT] care you provide:

1 2 3 4 5 Clinical data

1 2 3 4 5 Your professional opinion

1 2 3 4 5 Input from client

1 2 3 4 5 Input from family members

1 2 3 4 5 Input from other health care providers

17. [If answer to question 15 is yes] Who else is typically involved in setting individualized goals with elderly home care clients related to the [nursing/OT/PT] care you provide? (Please choose all that apply)  Client

Client’s family members (if applicable)

CCAC case manager

Home health care providers from my organization (if applicable)

Home health care providers from other organizations (if applicable)

Others (Please list) __________________________________________________________________________________________________________________________________________________________________________________________________________________________________________

Prefer not to say

18. [If answer to question 15 is yes] How do you record individual client goals? (Please choose all that apply)

I write them down and leave them in a file in the client’s home

I record them electronically in the client’s chart that my organization has established

I write them down in my own notes on the client that I keep with me

My clients and I verbally discuss their goals at each visit

Other (Please list) __________________________________________________________________________________________________________________________________________________________________________________________________________________________________________

Prefer not to say

19. Please describe any strategies you use to involve the client and family caregiver(s) in their care planning and decision-making. (Please type response in space below)

______________________________________________________________________________________________________________________________________________________________________________________________________________________________________________________________________________________________________________________________________________________________________________________________________

**Section 5: Interdisciplinary Collaboration**

20. Do you routinely directly share any information you collect about elderly home care clients with other health care providers involved in their care?

Yes

No

Prefer not to say

21. [If answer to question 20 is yes] Who do you routinely directly share information with related to an individual elderly home care client’s care? (Please choose all that apply)

Nurses within my organization

Occupational Therapists within my organization

Physiotherapists within my organization

Nurses from other home care organizations (involved in client’s care)

Occupational Therapists from other home care organizations (involved in client’s care)

Physiotherapists from other organizations (involved in client’s care)

Personal Support Workers (PSWs)

Social Workers

Speech Language Pathologists

Physicians

CCAC case managers

Other(s) (Please list) __________________________________________________________________________________________________________________________________________________________________________________________________________________________________________

Prefer not to say

22. [If answer to question 20 is yes] What information do you routinely share with other health care providers? (Please choose all that apply)

Clinical data

Client goals

Information about client’s environment

Information about client’s family

Details about client progress, lack of progress, revised care plans etc.

Other (please list) __________________________________________________________________________________________________________________________________________________________________________________________________________________________________________

23. [If answer to question 20 is no] Why do you not share information you collect about elderly home care clients with other health care providers involved in their care? (Please choose all that apply from the list below)

Privacy issues

I’m not usually aware of who the other health providers are

Other (please list)

__________________________________________________________________________________________________________________________________________________________________________________________________________________________________________

Prefer not to say

24. Do you routinely directly receive any information about elderly home care clients from other health care providers involved in their care?

Yes

No

Prefer not to say

25. [If answer to question 24 is yes] Who do you routinely receive information from related to an individual elderly home care client’s care? (Please choose all that apply)

Nurses within my organization

Occupational Therapists within my organization

Physiotherapists within my organization

Nurses from other home care organizations (involved in client’s care)

Occupational Therapists from other home care organizations (involved in client’s care)

Physiotherapists from other organizations (involved in client’s care)

Personal Support Workers (PSWs)

Social Workers

Speech Language Pathologists

Physicians

CCAC case managers

Other(s) (Please list) __________________________________________________________________________________________________________________________________________________________________________________________________________________________________________

Prefer not to say

26. [If answer to question 24 is yes] What information do you routinely receive from other health care providers? (Please choose all that apply)

Clinical data

Client goals

Information about client’s environment

Information about client’s family

Details about client progress, lack of progress, revised care plans etc.

27. [If answer to question 24 is no] Why do you not receive information you collect about elderly home care clients from other health care providers involved in their care? (Please choose all that apply from the list below)

Privacy issues

I’m not usually aware of who the other health providers are

Other (please list)

__________________________________________________________________________________________________________________________________________________________________________________________________________________________________________

Prefer not to say
