# Supplementary material for: Development and testing of the Geriatric Care Assessment Practices (G-CAP) survey
Source: BMC Geriatr. 2021 Apr 1;21:220. doi: 10.1186/s12877-021-02073-5 (PMC8015173; doi:10.1186/s12877-021-02073-5)
Supplement: Supplementary file 2 — Additional file 2. Pilot Version of the G-CAP Survey [file 12877_2021_2073_MOESM2_ESM.docx]

**Additional file 2- Pilot Version of the Geriatric Care Assessment Practices (G-CAP) Survey**

**Section 1: Methods of Assessment**

First, we would like to ask you some questions about your assessment practices with older adults during your first visit in their home.

***Cognition and Mood***

1. How often do you conduct an assessment (formal or informal) of an older adult’s cognition and mood during your first home care visit with them in order to make decisions about their care needs and/or provide care?

| ☐  Never | ☐  Almost never | ☐  Rarely | ☐ Sometimes | ☐  Often | ☐  Almost always | ☐  Always |
| --- | --- | --- | --- | --- | --- | --- |

***[If they answer Never, Almost Never or Rarely, fluid surveys will skip to question 3; if they answer Sometimes, Often, Almost Always or Always, fluid surveys will proceed to question 2]***

How often do you use the following assessment tools/approaches to collect information about an older home care client’s cognition and/or mood?

2a Montreal Cognitive Assessment (MoCA)

|  |  |  |  |  |  |  |
| --- | --- | --- | --- | --- | --- | --- |
| ☐  Never | ☐  Almost never | ☐  Rarely | ☐ Sometimes | ☐  Often | ☐  Almost always | ☐  Always |

2b Mini-Mental State Examination (MMSE)

| ☐  Never | ☐  Almost never | ☐  Rarely | ☐ Sometimes | ☐  Often | ☐  Almost always | ☐  Always |
| --- | --- | --- | --- | --- | --- | --- |

2c Centre for Epidemiological Studies Depression Scale (CES-D)

| ☐  Never | ☐  Almost never | ☐  Rarely | ☐ Sometimes | ☐  Often | ☐  Almost always | ☐  Always |
| --- | --- | --- | --- | --- | --- | --- |

2d Geriatric Depression Scale

| ☐  Never | ☐  Almost never | ☐  Rarely | ☐ Sometimes | ☐  Often | ☐  Almost always | ☐  Always |
| --- | --- | --- | --- | --- | --- | --- |

2e Glasgow Coma Scale

| ☐  Never | ☐  Almost never | ☐  Rarely | ☐ Sometimes | ☐  Often | ☐  Almost always | ☐  Always |
| --- | --- | --- | --- | --- | --- | --- |

2f The Delirium Index

| ☐  Never | ☐  Almost never | ☐  Rarely | ☐ Sometimes | ☐  Often | ☐  Almost always | ☐  Always |
| --- | --- | --- | --- | --- | --- | --- |

2g The Confusion Assessment Method (CAM)

| ☐  Never | ☐  Almost never | ☐  Rarely | ☐ Sometimes | ☐  Often | ☐  Almost always | ☐  Always |
| --- | --- | --- | --- | --- | --- | --- |

2h Delirium Rating Scale

| ☐  Never | ☐  Almost never | ☐  Rarely | ☐ Sometimes | ☐  Often | ☐  Almost always | ☐  Always |
| --- | --- | --- | --- | --- | --- | --- |

2i I use my own observation and/or interview skills to assess an older adult home care client’s cognition and mood

| ☐  Never | ☐  Almost never | ☐  Rarely | ☐ Sometimes | ☐  Often | ☐  Almost always | ☐  Always |
| --- | --- | --- | --- | --- | --- | --- |

***Pain***

3 How often do you conduct an assessment (formal or informal) of an older adult’s pain during your first home care visit with them in order to make decisions about their care needs and/or provide care?

| ☐  Never | ☐  Almost never | ☐  Rarely | ☐ Sometimes | ☐  Often | ☐  Almost always | ☐  Always |
| --- | --- | --- | --- | --- | --- | --- |

***[If they answer Never, Almost Never or Rarely, fluid surveys will skip to question 5; if they answer Sometimes, Often, Almost Always or Always, fluid surveys will proceed to question 4]***

How often do you use the following assessment tools/ approaches to collect information about an older home care client’s pain (area and intensity)?

4a Brief Pain Inventory

| ☐  Never | ☐  Almost never | ☐  Rarely | ☐ Sometimes | ☐  Often | ☐  Almost always | ☐  Always |
| --- | --- | --- | --- | --- | --- | --- |

4b Numeric Pain Rating Scale (NPRS)

| ☐  Never | ☐  Almost never | ☐  Rarely | ☐ Sometimes | ☐  Often | ☐  Almost always | ☐  Always |
| --- | --- | --- | --- | --- | --- | --- |

4c Verbal Rating Scale (Pain)

| ☐  Never | ☐  Almost never | ☐  Rarely | ☐ Sometimes | ☐  Often | ☐  Almost always | ☐  Always |
| --- | --- | --- | --- | --- | --- | --- |

4d Baker-Wong Pain Scale

| ☐  Never | ☐  Almost never | ☐  Rarely | ☐ Sometimes | ☐  Often | ☐  Almost always | ☐  Always |
| --- | --- | --- | --- | --- | --- | --- |

4e Visual Analogue Scale for Pain

| ☐  Never | ☐  Almost never | ☐  Rarely | ☐ Sometimes | ☐  Often | ☐  Almost always | ☐  Always |
| --- | --- | --- | --- | --- | --- | --- |

4f Facial Grimace and Behaviour Checklist Flowcharts (Pain)

| ☐  Never | ☐  Almost never | ☐  Rarely | ☐ Sometimes | ☐  Often | ☐  Almost always | ☐  Always |
| --- | --- | --- | --- | --- | --- | --- |

4g McGill Pain Questionnaire

| ☐  Never | ☐  Almost never | ☐  Rarely | ☐ Sometimes | ☐  Often | ☐  Almost always | ☐  Always |
| --- | --- | --- | --- | --- | --- | --- |

4h Northern Pain Scale

| ☐  Never | ☐  Almost never | ☐  Rarely | ☐ Sometimes | ☐  Often | ☐  Almost always | ☐  Always |
| --- | --- | --- | --- | --- | --- | --- |

4i I use my own observation and/or interview skills to assess an older adult home care client’s pain

| ☐  Never | ☐  Almost never | ☐  Rarely | ☐ Sometimes | ☐  Often | ☐  Almost always | ☐  Always |
| --- | --- | --- | --- | --- | --- | --- |

***Skin Integrity***

5 How often do you conduct an assessment (formal or informal) of an older adult’s skin integrity during your first home care visit with them in order to make decisions about their care needs and/or provide care?

| ☐  Never | ☐  Almost never | ☐  Rarely | ☐ Sometimes | ☐  Often | ☐  Almost always | ☐  Always |
| --- | --- | --- | --- | --- | --- | --- |

***[If they answer Never, Almost Never or Rarely, fluid surveys will skip to question 7; if they answer Sometimes, Often, Almost Always or Always, fluid surveys will proceed to question 6]***

How often do you use the following assessment tools/ approaches to collect information about an older home care patient’s skin integrity?

6a Braden Scale for Predicting Pressure Sore Risk

| ☐  Never | ☐  Almost never | ☐  Rarely | ☐ Sometimes | ☐  Often | ☐  Almost always | ☐  Always |
| --- | --- | --- | --- | --- | --- | --- |

6b Bates-Jensen Wound Assessment Tool (BWAT)

| ☐  Never | ☐  Almost never | ☐  Rarely | ☐ Sometimes | ☐  Often | ☐  Almost always | ☐  Always |
| --- | --- | --- | --- | --- | --- | --- |

6c Pressure Ulcer Scale for Healing (PUSH)

| ☐  Never | ☐  Almost never | ☐  Rarely | ☐ Sometimes | ☐  Often | ☐  Almost always | ☐  Always |
| --- | --- | --- | --- | --- | --- | --- |

6d I use my own observation and/or interview skills to assess an older adult home care client’s skin integrity

| ☐  Never | ☐  Almost never | ☐  Rarely | ☐ Sometimes | ☐  Often | ☐  Almost always | ☐  Always |
| --- | --- | --- | --- | --- | --- | --- |

***Functional Status/Activity and Rest***

7 How often do you conduct an assessment (formal or informal) of an older adult’s functional status/ activity and rest during your first home care visit with them in order to make decisions about their care needs and/or provide care?

| ☐  Never | ☐  Almost never | ☐  Rarely | ☐ Sometimes | ☐  Often | ☐  Almost always | ☐  Always |
| --- | --- | --- | --- | --- | --- | --- |

***[If they answer Never, Almost Never or Rarely, fluid surveys will skip to question 9; if they answer Sometimes, Often, Almost Always or Always, fluid surveys will proceed to question 8]***

How often do you use the following assessment tools to collect information about an older home care client’s functional status/ activity and rest?

8a Functional Independence Measure (FIM)

| ☐  Never | ☐  Almost never | ☐  Rarely | ☐ Sometimes | ☐  Often | ☐  Almost always | ☐  Always |
| --- | --- | --- | --- | --- | --- | --- |

8b Katz Index of Independence in Activities of Daily Living

| ☐  Never | ☐  Almost never | ☐  Rarely | ☐ Sometimes | ☐  Often | ☐  Almost always | ☐  Always |
| --- | --- | --- | --- | --- | --- | --- |

8c Barthel Index

| ☐  Never | ☐  Almost never | ☐  Rarely | ☐ Sometimes | ☐  Often | ☐  Almost always | ☐  Always |
| --- | --- | --- | --- | --- | --- | --- |

8d Borg Rating Scale of Perceived Exertion

| ☐  Never | ☐  Almost never | ☐  Rarely | ☐ Sometimes | ☐  Often | ☐  Almost always | ☐  Always |
| --- | --- | --- | --- | --- | --- | --- |

8e (SMAF) Functional Autonomy Measurement System

| ☐  Never | ☐  Almost never | ☐  Rarely | ☐ Sometimes | ☐  Often | ☐  Almost always | ☐  Always |
| --- | --- | --- | --- | --- | --- | --- |

8f Functional Reach Test

| ☐  Never | ☐  Almost never | ☐  Rarely | ☐ Sometimes | ☐  Often | ☐  Almost always | ☐  Always |
| --- | --- | --- | --- | --- | --- | --- |

8g OARS-IADL (Older Americans Resources and Services Scale-Instrumental Activities of Daily Living)

| ☐  Never | ☐  Almost never | ☐  Rarely | ☐ Sometimes | ☐  Often | ☐  Almost always | ☐  Always |
| --- | --- | --- | --- | --- | --- | --- |

8h Reintegration to Normal Living Index (RNL)

| ☐  Never | ☐  Almost never | ☐  Rarely | ☐ Sometimes | ☐  Often | ☐  Almost always | ☐  Always |
| --- | --- | --- | --- | --- | --- | --- |

8i (TEMPA) Test d’Evaluation des Membres Superieurs des Personnes Agees

| ☐  Never | ☐  Almost never | ☐  Rarely | ☐ Sometimes | ☐  Often | ☐  Almost always | ☐  Always |
| --- | --- | --- | --- | --- | --- | --- |

8j Canadian Occupational Therapy Performance Measure (COPM)

| ☐  Never | ☐  Almost never | ☐  Rarely | ☐ Sometimes | ☐  Often | ☐  Almost always | ☐  Always |
| --- | --- | --- | --- | --- | --- | --- |

8k Assessment of Motor and Process Skills (AMPS)

| ☐  Never | ☐  Almost never | ☐  Rarely | ☐ Sometimes | ☐  Often | ☐  Almost always | ☐  Always |
| --- | --- | --- | --- | --- | --- | --- |

8l I use my own observation/interview skills to assess an older adult home care client’s functional status/ activity and rest

| ☐  Never | ☐  Almost never | ☐  Rarely | ☐ Sometimes | ☐  Often | ☐  Almost always | ☐  Always |
| --- | --- | --- | --- | --- | --- | --- |

***Mobility/ Balance/ Ambulation***

9 How often do you conduct an assessment (formal or informal) of an older adult’s mobility/ balance/ ambulation during your first home care visit with them in order to make decisions about their care needs and/or provide care?

| ☐  Never | ☐  Almost never | ☐  Rarely | ☐ Sometimes | ☐  Often | ☐  Almost always | ☐  Always |
| --- | --- | --- | --- | --- | --- | --- |

***[If they answer Never, Almost Never or Rarely, fluid surveys will skip to question 11; if they answer Sometimes, Often, Almost Always or Always, fluid surveys will proceed to question 10]***

How often do you use the following assessment tools to collect information about an older home care client’s mobility/ balance/ ambulation?

10a Community Balance and Mobility Scale

| ☐  Never | ☐  Almost never | ☐  Rarely | ☐ Sometimes | ☐  Often | ☐  Almost always | ☐  Always |
| --- | --- | --- | --- | --- | --- | --- |

10b Lower Extremity Functional Scale

| ☐  Never | ☐  Almost never | ☐  Rarely | ☐ Sometimes | ☐  Often | ☐  Almost always | ☐  Always |
| --- | --- | --- | --- | --- | --- | --- |

10c Berg Balance Scale

| ☐  Never | ☐  Almost never | ☐  Rarely | ☐ Sometimes | ☐  Often | ☐  Almost always | ☐  Always |
| --- | --- | --- | --- | --- | --- | --- |

10d Short Form Berg Balance Scale 3 Point

| ☐  Never | ☐  Almost never | ☐  Rarely | ☐ Sometimes | ☐  Often | ☐  Almost always | ☐  Always |
| --- | --- | --- | --- | --- | --- | --- |

10e Timed Get Up and Go Test (TUG)

| ☐  Never | ☐  Almost never | ☐  Rarely | ☐ Sometimes | ☐  Often | ☐  Almost always | ☐  Always |
| --- | --- | --- | --- | --- | --- | --- |

10f Timed-Stands Test

| ☐  Never | ☐  Almost never | ☐  Rarely | ☐ Sometimes | ☐  Often | ☐  Almost always | ☐  Always |
| --- | --- | --- | --- | --- | --- | --- |

10g Five Times Sit to Stand Test

| ☐  Never | ☐  Almost never | ☐  Rarely | ☐ Sometimes | ☐  Often | ☐  Almost always | ☐  Always |
| --- | --- | --- | --- | --- | --- | --- |

10h Walk Test—2 minute, 6 minute, 12 minute, self-paced, shuttle

| ☐  Never | ☐  Almost never | ☐  Rarely | ☐ Sometimes | ☐  Often | ☐  Almost always | ☐  Always |
| --- | --- | --- | --- | --- | --- | --- |

10i Gait Speed

| ☐  Never | ☐  Almost never | ☐  Rarely | ☐ Sometimes | ☐  Often | ☐  Almost always | ☐  Always |
| --- | --- | --- | --- | --- | --- | --- |

10j Physiotherapy Functional Mobility Profile (PFMP)

| ☐  Never | ☐  Almost never | ☐  Rarely | ☐ Sometimes | ☐  Often | ☐  Almost always | ☐  Always |
| --- | --- | --- | --- | --- | --- | --- |

10k Activities-Specific Balance Confidence Scale

| ☐  Never | ☐  Almost never | ☐  Rarely | ☐ Sometimes | ☐  Often | ☐  Almost always | ☐  Always |
| --- | --- | --- | --- | --- | --- | --- |

10l I use my own observation and/or interview skills to assess an older adult home care client’s mobility/balance/ambulation

| ☐  Never | ☐  Almost never | ☐  Rarely | ☐ Sometimes | ☐  Often | ☐  Almost always | ☐  Always |
| --- | --- | --- | --- | --- | --- | --- |

***Safety (Environment, Abuse risk and Falls Risk)***

11 How often do you conduct an assessment (formal or informal) of an older adult’s safety (environment, abuse risk and falls risk) during your first home care visit with them in order to make decisions about their care needs and/or provide care?

| ☐  Never | ☐  Almost never | ☐  Rarely | ☐ Sometimes | ☐  Often | ☐  Almost always | ☐  Always |
| --- | --- | --- | --- | --- | --- | --- |

***[If they answer Never, Almost Never or Rarely, fluid surveys will skip to question 13; if they answer Sometimes, Often, Almost Always or Always, fluid surveys will proceed to question 12]***

How often do you use the following assessment tools/ approaches to collect information about an older home care client’s safety (environment, abuse risk and falls risk)?

12a SAFER-HOME

| ☐  Never | ☐  Almost never | ☐  Rarely | ☐ Sometimes | ☐  Often | ☐  Almost always | ☐  Always |
| --- | --- | --- | --- | --- | --- | --- |

12b Falls Risk Assessment Tool (FRAT)

| ☐  Never | ☐  Almost never | ☐  Rarely | ☐ Sometimes | ☐  Often | ☐  Almost always | ☐  Always |
| --- | --- | --- | --- | --- | --- | --- |

12c Falls Risk for Older People in the Community (FROP-Com)

| ☐  Never | ☐  Almost never | ☐  Rarely | ☐ Sometimes | ☐  Often | ☐  Almost always | ☐  Always |
| --- | --- | --- | --- | --- | --- | --- |

12d Indicators of Abuse (IOA)

| ☐  Never | ☐  Almost never | ☐  Rarely | ☐ Sometimes | ☐  Often | ☐  Almost always | ☐  Always |
| --- | --- | --- | --- | --- | --- | --- |

12e Caregiver Abuse Screen (CASE)

| ☐  Never | ☐  Almost never | ☐  Rarely | ☐ Sometimes | ☐  Often | ☐  Almost always | ☐  Always |
| --- | --- | --- | --- | --- | --- | --- |

12f I use my own observation and/or interview skills to assess an older adult home care client’s safety (environment, abuse risk and falls risk)

| ☐  Never | ☐  Almost never | ☐  Rarely | ☐ Sometimes | ☐  Often | ☐  Almost always | ☐  Always |
| --- | --- | --- | --- | --- | --- | --- |

***Quality of Life***

13 How often do you conduct an assessment (formal or informal) of an older adult’s quality of life during your first home care visit with them in order to make decisions about their care needs and/or provide care?

| ☐  Never | ☐  Almost never | ☐  Rarely | ☐ Sometimes | ☐  Often | ☐  Almost always | ☐  Always |
| --- | --- | --- | --- | --- | --- | --- |

***[If they answer Never, Almost Never or Rarely, fluid surveys will skip to question 15; if they answer Sometimes, Often, Almost Always or Always, fluid surveys will proceed to question 14]***

How often do you use the following assessment tools to collect information about an older home care client’s quality of life?

14a Community Integration Questionnaire II

| ☐  Never | ☐  Almost never | ☐  Rarely | ☐ Sometimes | ☐  Often | ☐  Almost always | ☐  Always |
| --- | --- | --- | --- | --- | --- | --- |

14b Life Satisfaction Questionnaire 9

| ☐  Never | ☐  Almost never | ☐  Rarely | ☐ Sometimes | ☐  Often | ☐  Almost always | ☐  Always |
| --- | --- | --- | --- | --- | --- | --- |

14c (EuroQoL-5D) European Quality of Life Scale

| ☐  Never | ☐  Almost never | ☐  Rarely | ☐ Sometimes | ☐  Often | ☐  Almost always | ☐  Always |
| --- | --- | --- | --- | --- | --- | --- |

14d Health Utilities Index (HUI Mark 2/3)

| ☐  Never | ☐  Almost never | ☐  Rarely | ☐ Sometimes | ☐  Often | ☐  Almost always | ☐  Always |
| --- | --- | --- | --- | --- | --- | --- |

14e Nottingham Health Profile

| ☐  Never | ☐  Almost never | ☐  Rarely | ☐ Sometimes | ☐  Often | ☐  Almost always | ☐  Always |
| --- | --- | --- | --- | --- | --- | --- |

14f SF-12 (12-item short-form health survey)

| ☐  Never | ☐  Almost never | ☐  Rarely | ☐ Sometimes | ☐  Often | ☐  Almost always | ☐  Always |
| --- | --- | --- | --- | --- | --- | --- |

14g SF-36 (Medical Outcomes Study 36-item short-form health survey)

| ☐  Never | ☐  Almost never | ☐  Rarely | ☐ Sometimes | ☐  Often | ☐  Almost always | ☐  Always |
| --- | --- | --- | --- | --- | --- | --- |

14h I use my own observation and/or interview skills to assess an older adult home care client’s quality of life

| ☐  Never | ☐  Almost never | ☐  Rarely | ☐ Sometimes | ☐  Often | ☐  Almost always | ☐  Always |
| --- | --- | --- | --- | --- | --- | --- |

***Medication Management***

15 How often do you conduct an assessment (formal or informal) of an older adult’s medication management during your first home care visit with them in order to make decisions about their care needs and/or provide care?

| ☐  Never | ☐  Almost never | ☐  Rarely | ☐ Sometimes | ☐  Often | ☐  Almost always | ☐  Always |
| --- | --- | --- | --- | --- | --- | --- |

***[If they answer Never, Almost Never or Rarely, fluid surveys will skip to question 17; if they answer Sometimes, Often, Almost Always or Always, fluid surveys will proceed to question 16]***

How often do you use the following assessment tools to collect information about an older home care client’s medication management?

16a Medication Management Ability Assessment

| ☐  Never | ☐  Almost never | ☐  Rarely | ☐ Sometimes | ☐  Often | ☐  Almost always | ☐  Always |
| --- | --- | --- | --- | --- | --- | --- |

16b Other tool (please list): ___________________________________

| ☐  Never | ☐  Almost never | ☐  Rarely | ☐ Sometimes | ☐  Often | ☐  Almost always | ☐  Always |
| --- | --- | --- | --- | --- | --- | --- |

16c I use my own observation and/or interview skills to assess an older adult home care client’s medication management

| ☐  Never | ☐  Almost never | ☐  Rarely | ☐ Sometimes | ☐  Often | ☐  Almost always | ☐  Always |
| --- | --- | --- | --- | --- | --- | --- |

***Resources (Social and Financial)***

17 How often do you conduct an assessment (formal or informal) of an older adult’s resources (social and financial) during your first home care visit with them in order to make decisions about their care needs and/or provide care?

| ☐  Never | ☐  Almost never | ☐  Rarely | ☐ Sometimes | ☐  Often | ☐  Almost always | ☐  Always |
| --- | --- | --- | --- | --- | --- | --- |

***[If they answer Never, Almost Never or Rarely, fluid surveys will skip to question 19; if they answer Sometimes, Often, Almost Always or Always, fluid surveys will proceed to question 18]***

How often do you use the following assessment tools/approaches to collect information about an older home care client’s resources (social and financial)?

18a Multidimensional Scale of Perceived Social Support

| ☐  Never | ☐  Almost never | ☐  Rarely | ☐ Sometimes | ☐  Often | ☐  Almost always | ☐  Always |
| --- | --- | --- | --- | --- | --- | --- |

18b Social Support Inventory (SSI)

| ☐  Never | ☐  Almost never | ☐  Rarely | ☐ Sometimes | ☐  Often | ☐  Almost always | ☐  Always |
| --- | --- | --- | --- | --- | --- | --- |

18c General Social Survey (GSS)

| ☐  Never | ☐  Almost never | ☐  Rarely | ☐ Sometimes | ☐  Often | ☐  Almost always | ☐  Always |
| --- | --- | --- | --- | --- | --- | --- |

18d Index of Social Support

| ☐  Never | ☐  Almost never | ☐  Rarely | ☐ Sometimes | ☐  Often | ☐  Almost always | ☐  Always |
| --- | --- | --- | --- | --- | --- | --- |

18e Practitioner Assessment of Network Type (PANT)

| ☐  Never | ☐  Almost never | ☐  Rarely | ☐ Sometimes | ☐  Often | ☐  Almost always | ☐  Always |
| --- | --- | --- | --- | --- | --- | --- |

18f Personal Resource Questionnaire (PRQ85)

| ☐  Never | ☐  Almost never | ☐  Rarely | ☐ Sometimes | ☐  Often | ☐  Almost always | ☐  Always |
| --- | --- | --- | --- | --- | --- | --- |

18g The MOS Social Support Survey

| ☐  Never | ☐  Almost never | ☐  Rarely | ☐ Sometimes | ☐  Often | ☐  Almost always | ☐  Always |
| --- | --- | --- | --- | --- | --- | --- |

18h The RAND Social Health Battery

| ☐  Never | ☐  Almost never | ☐  Rarely | ☐ Sometimes | ☐  Often | ☐  Almost always | ☐  Always |
| --- | --- | --- | --- | --- | --- | --- |

18i Assessment of Perceived Loneliness

| ☐  Never | ☐  Almost never | ☐  Rarely | ☐ Sometimes | ☐  Often | ☐  Almost always | ☐  Always |
| --- | --- | --- | --- | --- | --- | --- |

18j Assessment of Social Isolation

| ☐  Never | ☐  Almost never | ☐  Rarely | ☐ Sometimes | ☐  Often | ☐  Almost always | ☐  Always |
| --- | --- | --- | --- | --- | --- | --- |

18k Social Support Questionnaire

| ☐  Never | ☐  Almost never | ☐  Rarely | ☐ Sometimes | ☐  Often | ☐  Almost always | ☐  Always |
| --- | --- | --- | --- | --- | --- | --- |

18l Interpersonal Support Evaluation List (ISEL)

| ☐  Never | ☐  Almost never | ☐  Rarely | ☐ Sometimes | ☐  Often | ☐  Almost always | ☐  Always |
| --- | --- | --- | --- | --- | --- | --- |

18m Semi-Structured Clinical Interview for Financial Capacity; SCIFC

| ☐  Never | ☐  Almost never | ☐  Rarely | ☐ Sometimes | ☐  Often | ☐  Almost always | ☐  Always |
| --- | --- | --- | --- | --- | --- | --- |

18n I use my own observation and/or interview skills to assess an older adult home care client’s resources (social and financial)

| ☐  Never | ☐  Almost never | ☐  Rarely | ☐ Sometimes | ☐  Often | ☐  Almost always | ☐  Always |
| --- | --- | --- | --- | --- | --- | --- |

**Section 2: Attitudes towards client assessment in home care**

Next, we would like to ask you some questions about your ideas about client assessment in home care

Please indicate the extent to which you agree/ disagree with the following statements about client assessment:

19a Client assessment involves collecting information about individuals using standardized tools

| ☐Completely disagree | ☐  Strongly disagree | ☐Somewhat disagree | ☐  Neither agree nor disagree | ☐ Somewhat agree | ☐  Strongly agree | ☐ Completely agree |
| --- | --- | --- | --- | --- | --- | --- |

19b Client assessment requires a conversation with the client

| ☐Completely disagree | ☐  Strongly disagree | ☐Somewhat disagree | ☐  Neither agree nor disagree | ☐ Somewhat agree | ☐  Strongly agree | ☐ Completely agree |
| --- | --- | --- | --- | --- | --- | --- |

19c Client assessment involves a conversation with the patient’s family caregiver(s) (assuming they have one)

| ☐Completely disagree | ☐  Strongly disagree | ☐Somewhat disagree | ☐  Neither agree nor disagree | ☐ Somewhat agree | ☐  Strongly agree | ☐ Completely agree |
| --- | --- | --- | --- | --- | --- | --- |

19d Client assessment involves a conversation with other health care providers in my discipline

| ☐Completely disagree | ☐  Strongly disagree | ☐Somewhat disagree | ☐  Neither agree nor disagree | ☐ Somewhat agree | ☐  Strongly agree | ☐ Completely agree |
| --- | --- | --- | --- | --- | --- | --- |

19e Client assessment involves a conversation with other health care providers outside of my discipline

| ☐Completely disagree | ☐  Strongly disagree | ☐Somewhat disagree | ☐  Neither agree nor disagree | ☐ Somewhat agree | ☐  Strongly agree | ☐ Completely agree |
| --- | --- | --- | --- | --- | --- | --- |

19f Client assessment requires observation of the client in their home environment

| ☐Completely disagree | ☐  Strongly disagree | ☐Somewhat disagree | ☐  Neither agree nor disagree | ☐ Somewhat agree | ☐  Strongly agree | ☐ Completely agree |
| --- | --- | --- | --- | --- | --- | --- |

19g Client assessment data should be recorded in a client’s chart

| ☐Completely disagree | ☐  Strongly disagree | ☐Somewhat disagree | ☐  Neither agree nor disagree | ☐ Somewhat agree | ☐  Strongly agree | ☐ Completely agree |
| --- | --- | --- | --- | --- | --- | --- |

19h Client assessment is an administrative practice

| ☐Completely disagree | ☐  Strongly disagree | ☐Somewhat disagree | ☐  Neither agree nor disagree | ☐ Somewhat agree | ☐  Strongly agree | ☐ Completely agree |
| --- | --- | --- | --- | --- | --- | --- |

19i Client assessment impacts how I deliver care to a client

| ☐Completely disagree | ☐  Strongly disagree | ☐Somewhat disagree | ☐  Neither agree nor disagree | ☐ Somewhat agree | ☐  Strongly agree | ☐ Completely agree |
| --- | --- | --- | --- | --- | --- | --- |

19j Client assessment is an ongoing process throughout a client’s care journey

| ☐Completely disagree | ☐  Strongly disagree | ☐Somewhat disagree | ☐  Neither agree nor disagree | ☐ Somewhat agree | ☐  Strongly agree | ☐ Completely agree |
| --- | --- | --- | --- | --- | --- | --- |

19k In order to provide care to a client, I must conduct the client assessment myself

| ☐Completely disagree | ☐  Strongly disagree | ☐Somewhat disagree | ☐  Neither agree nor disagree | ☐ Somewhat agree | ☐  Strongly agree | ☐ Completely agree |
| --- | --- | --- | --- | --- | --- | --- |

19l I can make use of client information collected by other health care professionals to provide care to clients

| ☐Completely disagree | ☐  Strongly disagree | ☐Somewhat disagree | ☐  Neither agree nor disagree | ☐ Somewhat agree | ☐  Strongly agree | ☐ Completely agree |
| --- | --- | --- | --- | --- | --- | --- |

**Section 3: Perceptions of the InterRAI-HC Assessment Tool**

Next, we would like to ask you some questions about your experience with and perceptions of the inter-RAI home care assessment tool (RAI-HC).

20 I have heard about the RAI-HC assessment tool before

| ☐  Yes | ☐  No |
| --- | --- |

***[If they answer yes, fluid surveys will proceed to question 21; if they answer no, fluid surveys will skill to question 23]***

Please indicate your level of experience with the RAI-HC

21a I use the RAI-HC to conduct comprehensive assessments of older home care clients

| ☐  Never | ☐  Almost never | ☐  Rarely | ☐ Sometimes | ☐  Often | ☐  Almost always | ☐  Always |
| --- | --- | --- | --- | --- | --- | --- |

21b I use RAI-HC data collected by someone else to plan and deliver care to older home care clients

| ☐  Never | ☐  Almost never | ☐  Rarely | ☐ Sometimes | ☐  Often | ☐  Almost always | ☐  Always |
| --- | --- | --- | --- | --- | --- | --- |

21c I use Clinical Assessment Protocols (CAPs) associated with the RAI-HC to plan and deliver care to older home care clients

| ☐  Never | ☐  Almost never | ☐  Rarely | ☐ Sometimes | ☐  Often | ☐  Almost always | ☐  Always |
| --- | --- | --- | --- | --- | --- | --- |

Please indicate the extent to which you agree/ disagree with the following statements about the RAI-HC :

22a The RAI-HC tool includes all the information I need to plan and deliver care to an older home care client

| ☐Completely disagree | ☐  Strongly disagree | ☐Somewhat disagree | ☐  Neither agree nor disagree | ☐ Somewhat agree | ☐  Strongly agree | ☐ Completely agree |
| --- | --- | --- | --- | --- | --- | --- |

22b The RAI-HC tool is too long to complete in a home care visit with an older home care client

| ☐Completely disagree | ☐  Strongly disagree | ☐Somewhat disagree | ☐  Neither agree nor disagree | ☐ Somewhat agree | ☐  Strongly agree | ☐ Completely agree |
| --- | --- | --- | --- | --- | --- | --- |

22c Collecting data with the RAI-HC is an administrative practice completed by the CCAC

| ☐Completely disagree | ☐  Strongly disagree | ☐Somewhat disagree | ☐  Neither agree nor disagree | ☐ Somewhat agree | ☐  Strongly agree | ☐ Completely agree |
| --- | --- | --- | --- | --- | --- | --- |

22d I feel confident that I can use the RAI-HC to collect information about an older home care client

| ☐Completely disagree | ☐  Strongly disagree | ☐Somewhat disagree | ☐  Neither agree nor disagree | ☐ Somewhat agree | ☐  Strongly agree | ☐ Completely agree |
| --- | --- | --- | --- | --- | --- | --- |

22e I feel confident that I can interpret data from the RAI-HC assessment to plan and deliver care to older home care clients

| ☐Completely disagree | ☐  Strongly disagree | ☐Somewhat disagree | ☐  Neither agree nor disagree | ☐ Somewhat agree | ☐  Strongly agree | ☐ Completely agree |
| --- | --- | --- | --- | --- | --- | --- |

22f RAI-HC data that gets collected by the CCAC is not linked to the care I provide

| ☐Completely disagree | ☐  Strongly disagree | ☐Somewhat disagree | ☐  Neither agree nor disagree | ☐ Somewhat agree | ☐  Strongly agree | ☐ Completely agree |
| --- | --- | --- | --- | --- | --- | --- |

**Section 4: Interdisciplinary Collaboration**

Next, we would like to ask you some questions about your experiences sharing and receiving information from other health care providers and working together to set individualized client goals.

Please indicate the relative importance of each of the following sources of input/ information for setting individual client goals in home care for older adults:

23a Assessment data that I collect

| ☐  Not at all important | ☐  Not very important | ☐ Somewhat un-important | ☐  Neutral | ☐ Important | ☐  Very Important | ☐ Extremely important |
| --- | --- | --- | --- | --- | --- | --- |

23b Assessment data that others collect

| ☐  Not at all important | ☐  Not very important | ☐ Somewhat un-important | ☐  Neutral | ☐ Important | ☐  Very Important | ☐ Extremely important |
| --- | --- | --- | --- | --- | --- | --- |

23c My professional opinion

| ☐  Not at all important | ☐  Not very important | ☐ Somewhat un-important | ☐  Neutral | ☐ Important | ☐  Very Important | ☐ Extremely important |
| --- | --- | --- | --- | --- | --- | --- |

23d The professional opinion of other health care providers

| ☐  Not at all important | ☐  Not very important | ☐ Somewhat un-important | ☐  Neutral | ☐ Important | ☐  Very Important | ☐ Extremely important |
| --- | --- | --- | --- | --- | --- | --- |

23e Information from the client’s chart

| ☐  Not at all important | ☐  Not very important | ☐ Somewhat un-important | ☐  Neutral | ☐ Important | ☐  Very Important | ☐ Extremely important |
| --- | --- | --- | --- | --- | --- | --- |

23f Input from the client

| ☐  Not at all important | ☐  Not very important | ☐ Somewhat un-important | ☐  Neutral | ☐ Important | ☐  Very Important | ☐ Extremely important |
| --- | --- | --- | --- | --- | --- | --- |

23g Input from the client’s family members

| ☐  Not at all important | ☐  Not very important | ☐ Somewhat un-important | ☐  Neutral | ☐ Important | ☐  Very Important | ☐ Extremely important |
| --- | --- | --- | --- | --- | --- | --- |

Please indicate the extent to which you agree/disagree with the following statements about your collaboration with others in the care of older home care patients

24a I always know what other health care providers are working with an older home care client I care for

| ☐Completely disagree | ☐  Strongly disagree | ☐Somewhat disagree | ☐  Neither agree nor disagree | ☐ Somewhat agree | ☐  Strongly agree | ☐ Completely agree |
| --- | --- | --- | --- | --- | --- | --- |

24b All health care providers involved in the care for an older home care client work towards common goals

| ☐Completely disagree | ☐  Strongly disagree | ☐Somewhat disagree | ☐  Neither agree nor disagree | ☐ Somewhat agree | ☐  Strongly agree | ☐ Completely agree |
| --- | --- | --- | --- | --- | --- | --- |

24c Privacy and confidentiality prevent me from sharing information about a client’s situation with other health care providers

| ☐Completely disagree | ☐  Strongly disagree | ☐Somewhat disagree | ☐  Neither agree nor disagree | ☐ Somewhat agree | ☐  Strongly agree | ☐ Completely agree |
| --- | --- | --- | --- | --- | --- | --- |

24d I feel like I am part of an integrated team when I am caring for older home care clients

| ☐Completely disagree | ☐  Strongly disagree | ☐Somewhat disagree | ☐  Neither agree nor disagree | ☐ Somewhat agree | ☐  Strongly agree | ☐ Completely agree |
| --- | --- | --- | --- | --- | --- | --- |

Please indicate how often you collaborate with other health care professionals to care for older home care clients in the following ways:

25a I share client assessment information with other home health care providers in my discipline

| ☐  Never | ☐  Almost never | ☐  Rarely | ☐ Sometimes | ☐  Often | ☐  Almost always | ☐  Always |
| --- | --- | --- | --- | --- | --- | --- |

25b I share client assessment information with home health care providers in other disciplines

| ☐  Never | ☐  Almost never | ☐  Rarely | ☐ Sometimes | ☐  Often | ☐  Almost always | ☐  Always |
| --- | --- | --- | --- | --- | --- | --- |

25c I share client assessment information with personal support workers (PSWs)

| ☐  Never | ☐  Almost never | ☐  Rarely | ☐ Sometimes | ☐  Often | ☐  Almost always | ☐  Always |
| --- | --- | --- | --- | --- | --- | --- |

25d I receive client assessment information from other home health care providers in my discipline

| ☐  Never | ☐  Almost never | ☐  Rarely | ☐ Sometimes | ☐  Often | ☐  Almost always | ☐  Always |
| --- | --- | --- | --- | --- | --- | --- |

25e I receive client assessment information from home health care providers outside of my discipline

| ☐  Never | ☐  Almost never | ☐  Rarely | ☐ Sometimes | ☐  Often | ☐  Almost always | ☐  Always |
| --- | --- | --- | --- | --- | --- | --- |

**Section 5: Demographic Information**

We are asking that you provide some demographic information so that we can describe the overall characteristics of the group of survey participants in our reports. This information will be reported as summary statistics only, will not be used at any time to identify you individually and will be kept strictly confidential at all times.

26 What is your gender identity?

☐Male

☐Female

27 What is your year of birth?

[drop down list]

28 What is your professional designation? (Please choose from the list below)

Registered Nurse

Registered Practical Nurse

Occupational Therapist

Physiotherapist

29 In what year did you receive this professional designation?

[drop down list]

30 How long have you been working in the home care sector? (Please choose from the list below)

Less than one year 1

1-5 years 2

6-10 years 3

Greater than 10 years 4

What other health care sectors have you worked in? (choose all that apply)

31a ☐Hospital

31b ☐In-patient rehabilitation

31c ☐Long-term care

31d☐Palliative care

31e☐Private sector

32 What home care provider agency(ies) do you currently work for? (choose all that apply)

[list names of participating organizations]

33 Approximately what percentage of the clients you work with in the community are over the age of 65?

Less than 25%

25-50%

51-75%

More than 75%
